# Ultrasound-Assisted Metal-Mediated Method for the Formation of Tetrahydro-3,3′-Disubstituted Biscoumarins

**DOI:** 10.3390/molecules23112810

**Published:** 2018-10-29

**Authors:** Ana I. Koleva, Nevena I. Petkova-Yankova, Rositca D. Nikolova

**Affiliations:** Department of Organic Chemistry and Pharmacognosy, Faculty of Chemistry and Pharmacy, Sofia University “St. Kliment Ohridski”, 1 J. Bouchier Buld., 1164 Sofia, Bulgaria; koleva.ana@gmail.com (A.I.K.), nipetkova@chem.uni-sofia.bg (N.I.P.-Y.)

**Keywords:** coumarins, biscoumarins, organometallic reagents, radical coupling, homodimerization, ultrasound irradiation, aci-nitro tautomer

## Abstract

A new method for faster and simple preparation of 3,3′,4,4′-tetrahydro-3,3′-disubstituted-4,4′-biscoumarins with participation of an organozinc reagent is reported. The reaction is promoted by ultrasound irradiation and it offers a simple experimental setup and excellent reproducibility of the results. Moreover, homodimers were isolated in yields of 45–92%. The dimerization conditions are applicable to coumarins with electron-withdrawing groups at third position.

## 1. Introduction

Benzopyrans and their analogs are a large class of compounds characterized by great diversity of pharmacological properties. The main members of this class of compounds are coumarins, 3,4-dihydrocoumarins, flavonoids, and biscoumarins isolated from different medical plants. These substances are synthesized by the plants themselves and have great biochemical significance for the proper functioning of individual plant parts.

Biscoumarins are a relatively unexplored class of compounds, isolated and characterized from different plant species [1]. It has been found that biscoumarins exhibit properties similar to those of coumarins and act as anticoagulants, antioxidants, antitumor, and antifungal agents. Essential and not well studied is their role as inhibitors of various enzymes—urease, anti-HIV-1 protease and integrase, DNA polymerase, and protein kinase [2,3,4,5,6].

A large number of biscoumarins and bis-3,4-dihydrocoumarins, regarding the type, lengths, and locations of the linkage, have been isolated and characterized. There are different dimerized coumarin systems of which 3,3′-, 4,4′-, 3,8′-, 3,6′-, 8,8′-, and 8,6′-biscoumarins are well known [7].

In reference to our ongoing work [8] for the possibility for conjugated addition of organometallic compounds to 3-substituted coumarins under ultrasound irradiation, we became interested in the synthesis of biscoumarin structures where two coumarin fragments of the same or different type are linked through C4-C4′ bond. The sonication method provides an unusual mechanism to generate high-energy chemistry due to the extraordinary temperatures and pressure generated by the cavitation bubble collapse [9,10,11,12], thus enhancing reactions involving radicals and radical ions. Therefore, the present paper reports the application of ultrasound irradiation in the formation of tetrahydroisomers of different 3,3′-disubstituted biscoumarins.

## 2. Results and Discussion

### 2.1. Synthesis of 3,3′,4,4′-Tetrahydro-3,3′-disubstituted-4,4′-biscoumarins

Different formation methods of biscoumarin structures are described in the literature. One of the commonly used methods for the formation of dimeric systems is by electrochemical reactions. The reduction of coumarins is accomplished by using different metal electrodes in the presence or absence of variety of catalysts (e.g., amines, alkaloids, etc.) [13,14,15,16,17,18,19,20]. The described electrochemical reactions are applicable to both unsubstituted coumarins and coumarins substituted by electron donor groups in 3-rd or 4-th position in the lactone ring. The formation of the dimeric systems is explained by the formation of radicals and radical anions.

Studies on the photochemically initiated reaction of coumarins and their 3,4-dihydro derivatives have reported radical formation where the dimer was identified as the major product. The substrates used were substituted coumarins with an acetyl and carboxylic group in 3-rd position [21,22,23].

The synthetic procedures for the formation of 4,4′-biscoumarins include the usage of the Wittig reaction where the two fragments can be formed simultaneously or one after another [24]. The formed dimeric structure resembles the structure of 4,4′-biisofraxidin that has been isolated from plants [25]. Later 4,4′-biisofraxidin was synthesized [26] in a multistep procedure with an overall yield of 7%. A Ni-catalyzed cross-coupling was applied in the last step of the study. A few years later the same authors modified the conditions for the Ullmann type cross-coupling reaction between coumarin fragments using a variety of leaving groups in 4-th position [27].

**Scheme 1:**
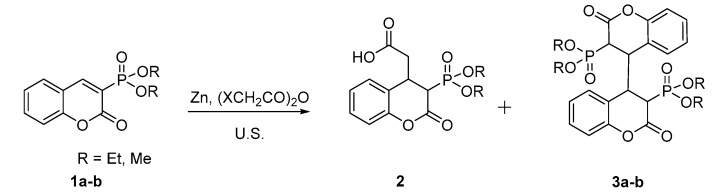
Dimeric compound of **1a**–**b**.

While trying to perform a 1,4-conjugate addition reaction to 3-diethylphosphonocoumarin **1a** using zinc and halogen anhydride under ultrasound conditions a dimeric dihydroisomer **3a** (Scheme 1) was isolated instead of the expected product **2**. Due to the structure of compound **3a** we became interested in obtaining tetrahydroisomers which could be synthesized in one step from 3-substituted coumarins under sonication. To design a synthetic strategy, we require high selectivity and quantitative yields. Firstly, we find the suitable conditions which ensure the formation of the dihydrocoumarin dimer as the only product. Secondly, we focus on reactions going smoothly with satisfactory yields. 3-Diethylphosphonocoumarin **1a** was chosen as a model compound due to the fact that the first formation of the dimer **3a** was observed when using it.

Grignard reagents are strongly basic and nucleophilic as it is known from the literature. Previous studies in our research group report [8,28,29] the 1,2- and 1,4-conjugate addition of organometallic compounds to 3-substituted coumarins. Even when the substituent is a phenyl group and the double bound of the coumarin system is not activated—1,4- and 1,2-addition adducts are observed.

Another intriguing fact is that Grignard reagents could be radical initiators in reactions with different compounds [30]. One of the first examples of producing coumarin radicals using *tert*-butylmagnesium chloride was the study of Gustafsson and coworkers [31] which presented the formation of bis-3,4-dihydrocoumarin from ethyl 3-coumarincarboxylate. Because of the complex reaction mixture and the low yield (1–10%) of tetrahydrobiscoumarin, its pure isolation was unsuccessful. The authors assumed that the observed results from the reaction could only be explained by radical formation in the transition state.

Regarding the utilized conditions and the known behavior of the coumarin substrates, the most probable path of the studied reaction could be assumed as a single-electron-transfer. Thus, the nature of the organometallic reagent was studied by comparing the applied metal—zinc or magnesium. The results showed equal conversion time in both cases but the yield of the desired product was only 14% for the organomagnesium and 70% for the organozinc reagent (see Table 1). The observed results have impact on the study of the organomagnesium reagents behavior during reactions with coumarins. Most probably the reduced amount of the homodimerization product is due to the small fraction of radicals leaving the magnesium surface. This conclusion is based on the surface nature of the Grignard reagent formation [30]. Therefore, organomagnesium reagents are better nucleophiles [8,28,29] than being suitable for initiation of coumarin radicals.

The organozinc compounds are relatively less reactive then RMgX, however the reactivity could be improved when chelating ligands are used. In the literature there are examples that present the formation of radicals by the assistance of the zinc compounds [32]. Based on the surface nature of the formation of organozinc compounds we suppose that when zinc is used the amount of the radicals that can escape the lattice is larger, resulting in the formation of the homodimerized product. Another advantage in the usage of RZnX for this type of reaction is the enhanced Lewis acidity of the zinc atom in RZnX, resulting in a stabilized radical transition state. This stabilization can be due to formation of a chelate complex with a donor molecule [33,34].

**Scheme 2:**
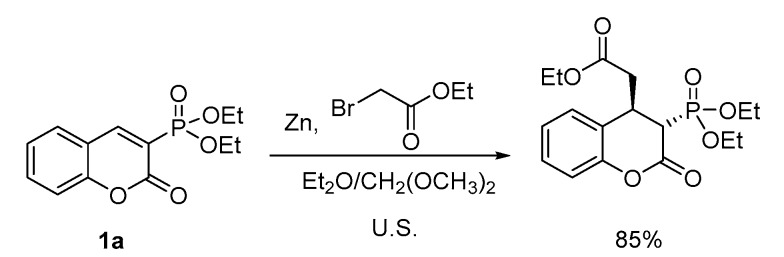
Reaction of **1a** with the Reformatsky reagent [8].

In our previous work [8] the formation of 1,4-addition product of organozinc reagent of ethylbromoacetate to the coumarin system under ultrasound irradiation conditions was observed (Scheme 2). Moreover, additional products or dimers were not observed in that study. Therefore, in the studied reaction conditions the organozinc reagent of chloroacetic anhydride is of a significant importance for the homodimerization of coumarins.

The reaction is sensitive to a number of effects and its outcome cannot be estimated easily. Common solvents for the organometallic reactions are diethyl ether and tetrahydrofuran (Et_2_O, THF). In the studied reaction THF was the solvent that accelerated the reaction rate with conversion time for compound **1a** of 2 h. However, we obtained a complex mixture rendering the separation of individual compounds impossible. The reaction did not occur in Et_2_O as a solvent (Table 2). A possible assumption for the observed results is the different solvation of the radicals formed in the transition state. It can be implied that THF is the most suitable solvent for single-electron-transfer reactions. Combining the two solvents, we managed to optimize the reaction rate. When the solvent ratio is THF: Et_2_O = 3.5:5 the reaction goes fluently for 7 h and the desired product is easily isolated with a yield of 70% using zinc as a metalorganic precursor.

The dimerization process for the tetrahydrobiscoumarin **3a** was optimized by reflux or ultrasound irradiation. In all the studied cases sonication has an advantage over thermal initiation, hence, we based our investigations are based on applying that technique. The reaction was carried out using different ratios of coumarin **1a**, metal, and chloroacetic anhydride. The reagent ratios and the reaction conditions are presented as Method A, B, and C in Table 1. The crucial distinction between the described methods is the amount of the applied zinc powder with respect to the anhydride.

A full conversion of **1a** was observed in 420 min when a slight excess of zinc was used (Method A). The addition of a double amount of zinc powder to the anhydride resulted in shorter reaction time and in increased yield of **3a** (Method B). To test the reaction rate, the metal to anhydride ratio was kept the same but the amount of reagents used was reduced (Method C) in aim to diminish the quantities. However, we did not observe a full conversion of the coumarin **1a** even though the reaction was carried for 24 h. Thus, we conclude that the preferred condition for homodimerization of 3-dialkylphosphonocoumarins **1a**–**b** is Method B.

**Scheme 3:**
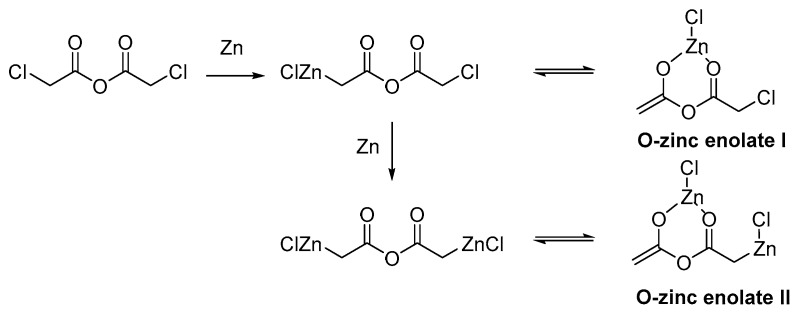
Structures of the possible organozinc enolates.

To explain our results from the reaction of **1a,** we consider two possible structures for the organozinc reagent. The small amount of zinc suggested a formation of monozinc compound which could be presented as C-zinc and O-zinc enolate given in Scheme 3. Therefore, the long reaction time might be due to the stabilization of the reagent by O-zinc enolate I complex that could react slowly in the implied conditions. When enough metal powder is presented in the reaction mixture there might be a possibility for forming a di-C-zinc enolate with chloroacetic anhydride. The dizinc reagent could as well be stabilized by an O-zinc enolate II, thus, leaving one end of the organozinc compound free to react with the coumarin system (Scheme 3).

**Scheme 4:**
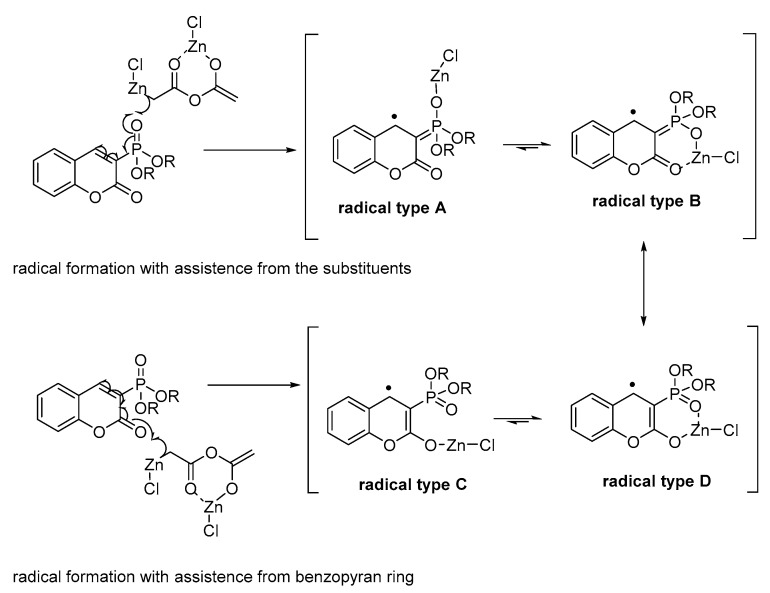
Possibilities for radical formation during the reaction.

These results suggest that the most probable reaction path for the coumarin **1a** includes radical initiation. The examples for radical formation in the presence of organometallic reagents have shown [31,35,36] a coordination of the organometallic reagent to the substrate with a single electron transfer. In case of substituted coumarin compounds there are two possible types of coordination. The first one includes the assistance of the carbonyl group from the benzopyran ring and the other—the substituents in third position. We assume that the initial step might be the formation of the coumarin “radical type A” or “radical type C” (Scheme 4). Both the adjacency of suitable groups with lone pairs and the presence of a metal, indicate a possibility for chelation in the radical with structural stabilization. Therefore, we suppose that, the two resonance structures—“radical type B” and “radical type D”—are more stable and are predominantly presented in the transition state.

**Scheme 5:**
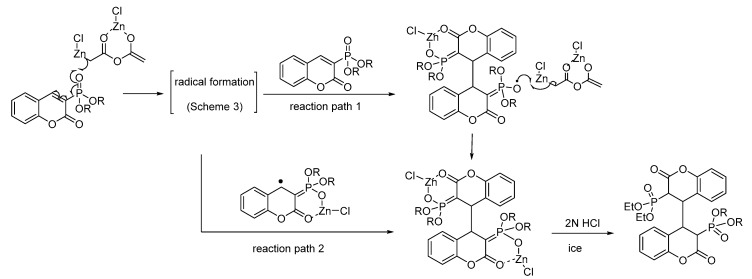
Probable mechanism for the formation of 3,3′,4,4′-tetrahydro-3,3′-disubstituted-4,4′-biscoumarins using 3-dialkylphosphocoumarins **1a**–**b** as model compounds.

Further analysis of the performed reaction indicates that once the radical generation takes place, there are two possible paths for the formation of bischromanes **3** from 3-diethylphosphonocoumarin **1a**, as illustrated in Scheme 5. The first path includes a radical Michael-type addition to a coumarin system, while the second path might involve a coupling of the initiated radicals [37].

**Scheme 6:**
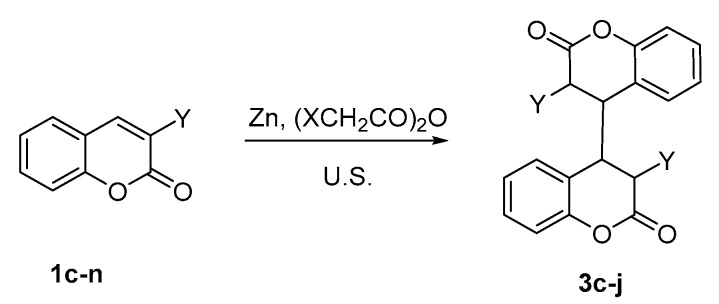
Homodimerization of coumarins **1c**–**n** under ultrasound irradiation.

The optimized reaction conditions from Method B were applied to a series of 3-substituted coumarins with different electron-withdrawing and electron-donating groups **1c**–**n** (Scheme 6) and tetrahydroisomers of 4,4′-biscoumarins **3c**–**j** were isolated with yields from 45 to 92% (see Table 3). Identically to the esters of 3-phosphonocoumarins **1a**–**b**, the esters of coumarin-3-carboxylic acid **1c**–**e** reacted smoothly and in shorter reaction times. The reaction with coumarins **1f**–**i** having a carbonyl group in third position was accomplished with the preparation of the all tetrahydrobiscoumarins **3f**–**i**. As shown in Table 3, we observe faster dimerization compared to coumarins carrying ester or phosphonoic group. However, the hindrance effect of these groups is crucial to radical initiation and reaction time. When the conditions of Method A were used for the dimerization reaction, a partial conversion of the substrate or complex mixtures for some of the coumarins were observed.

**Scheme 7:**
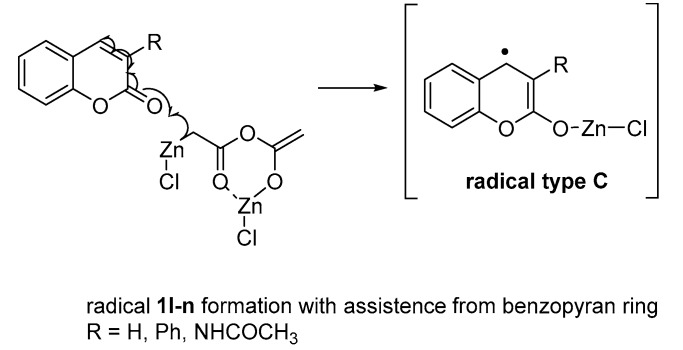
Structures of a possible radicals type C for compounds **1l**–**n**.

Interestingly, in cases of coumarins **1l**–**n** no interaction was observed under ultrasound irradiation although 2-oxochromane **1l** participates in electrochemical reactions showing excellent yield for tetrahydrocoumarin. Analyzing the results, we assume that if the radical formation occurs with the assistance of the carbonyl group of the pyran ring we should observe the formation of **3l**–**n** because the formation of radical type C (Scheme 7) is not affected by the substituents in 3-rd position. Though, the dimeric product might be with lower yield due to the less stability of the formed radical. The findings during the investigations on compounds **1l**–**n** incontestably illustrate the impossibility of coumarins with electron-donating groups to participate in the described homodimerization conditions. Thus, the assistance of the electron-withdrawing substituent is crucial for the initiation step and further stabilization of the radical as a chelated complex.

The reactions with coumarins **1j** and **1k** were carried out applying Method B resulting in full conversion. However, only bisoxochromane of 3-nitrocoumarin **3j** was characterized after purification with column chromatography. In the case of coumarin **1k**, out of many products detected by TLC in the reaction mixture, not one corresponds to a biscoumarin structure. Applying Method A none of the dimeric structures were isolated due to the absence of predominant product.

Regarding the mechanism of coumarin dimerization, another approach was utilized to highlight the behavior of initiated radicals in the reaction mixture. Three approaches for a mixed reaction were chosen comparing coumarin systems with different time for activation or radical formation and their capability to participate in the studied reaction conditions. The reactions were monitored by TLC and ^1^H NMR. Firstly, coumarins **1a** and **1m** were mixed in equimolar ratio under the conditions of Method B (Table 4, entry 1) and after three h only the homodimer **3a** was isolated. The result was unexpected but it revised our expectation for Michael type addition to the coumarin conjugated system and directed the options towards the radical coupling. The next experiment (Table 4, entry 2) illustrated the properties of two reactive coumarins—**1a** and **1c**—and, for a second time, we observed a dimerization of radicals from the same type. In this entry for a short time (40 min), a conversion of **1c** was monitored and the biscoumarin **3c** was detected. At the end (Table 4, entry 3), a reaction between **1f** and **1i** was carried out as an example of two systems giving dimers in a comparable conversion time in the applied conditions. The yields and the reaction time of coumarins **1f** and **1i** made them the most suitable candidates for testing our predictions. The full conversion of the starting compounds and the registration of the products **3f** and **3i** had supported the idea of a process that favors the radical homocoupling at the moment of initiation on the metal surface.

### 2.2. Spectroscopic Data Interpretation of Homodimers ***3***

The structure of the dimeric compound **3** was mainly analyzed by 1D and 2D NMR spectroscopy. The data for the 3,3′,4,4′-tetrahydro-3,3′-disubstituted-4,4′-biscoumarins present a symmetric structure for all of the compounds. The knowledge on this type of compounds have also demonstrated similar 4,4′-bis-2-oxochromane structures of the dimeric coumarin systems [14,16,19].

Keeping in mind the presence of four stereocenters in the structure of **3** it is normal to expect several stereoisomers. However, the tetrahydrobiscoumarins **3a**–**e** produced from esters of coumarin-3-carboxylic acid and dialkyl phosphonoesters appear as a single isomer. The signals of H-3 and H-4 protons in ^1^H NMR of **3c**–**e** are singlets without measurable *J*-constants. This means the position of the two nuclei determine a very small coupling between them or a constant with value less than 0.9 Hz. The location of the protons could mean 90 degrees between H-3 and H-4. Furthermore, it points to an antiperiplanar disposition of the bulky substituents around the C3-C4 bond. The same conformation was found in the products of conjugate addition of organometallic reagents to 3-substituted coumarins under ultrasound irradiation in our previous study [8]. Protons H-3 and H-4 appeared as doublets in compounds **3a**–**b** with high phosphorus–proton coupling constant. The two characteristic protons were assigned from the HMBC spectra for all tetrahydrobiscoumarins **3**.

**Figure 1:**
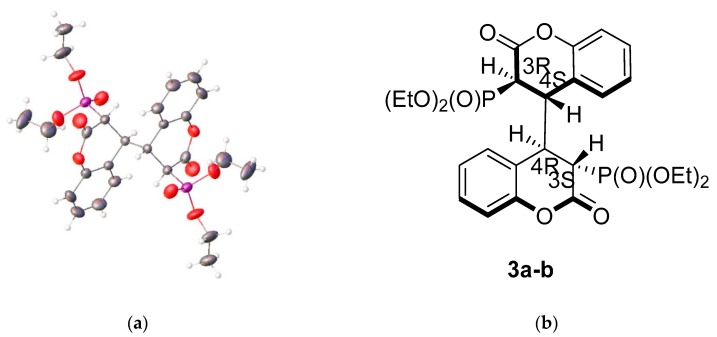
Structure of compound **3a**: (**a**) ORTEP representation with thermal ellipsoids 40%—Crystal Data for C_26_H_32_O_10_P_2_ (*M* = 566.45 g/mol): monoclinic, space group P2_1_/n (no. 14), *a* = 12.3959(15) Å, *b* = 9.3824(10) Å, *c* = 12.4119(16) Å, β = 101.126(5)°, *V* = 1416.4(3) Å^3^, *Z* = 2, *T* = 300.15 K, μ(MoKα) = 0.207 mm^−1^, *Dcalc* = 1.328 g/cm^3^, 9883 reflections measured (5.17° ≤ 2Θ ≤ 52.788°), 2888 unique (*R*_int_ = 0.0528, R_sigma_ = 0.0587) which were used in all calculations. The final *R*_1_ was 0.0507 (I > 2σ(I)) and *wR*_2_ was 0.1269 (all data). (**b**) *Meso*-form of compound **3a**.

The structure proposed by NMR spectra was confirmed with a single crystal X-ray structure of homodimer **3a** (CCDC1858604, Figure 1a, see also Supporting Materials). Based on the gathered results, we assume that the only isomer to be expected in cases **3a**–**e** is the (3R,4S,3′S,4′R)-stereoisomer or the *meso*-form where the elements of symmetry minimized the numbers of isomers. Thus, the stereoselectivity of the homodimerization reaction is determined by the steric hindrance of the substituent in position 3 in the pyran ring. Additional evidence for the absence of another diastereomer is the singlet in the ^31^P NMR spectra of **3a** and **3b** according to external referent standard H_3_PO_4_.

**Figure 2:**
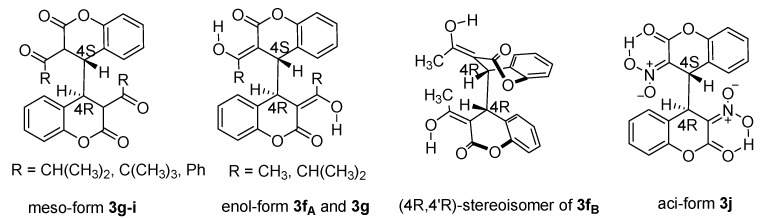
Stereoisomers of tetrahydrobiscoumarins **3g**–**j**.

Data from the NOESY spectra for the all *meso*-dimers demonstrate the correlation between protons H-5/H-5′ from one of the benzene ring with H-3′/H-3 and protons from the substituent in position 3. Another close correlation observed is between H-5 and H-4 from one of the 2-oxo-2*H*-chromane fragments. These findings supported a stereoisomer in *meso*-form having formally presented *s*-*trans* alignment of the benzopyran fragments around C4-C4′ bond (Figure 2).

Difficulties in the stereoselectivity of the reaction arose when ketones were implied due to the small steric hindrance of the substituent and the adjacency of an alpha-proton to the C=O group. While analyzing the NMR spectra for the products of coumarins **1f**–**i** we found two isomers with the tetrahydrobiscoumarin structure as well as enol-forms of the stereoisomers in cases of 3-acetyl- and 3-isopropylcoumarins. The tendency for keto-enol tautomerism of the products of 3-acetylcoumarin is well-known to us [38,39] and also noted in many examples in the literature even for bis-2-oxochromanes [21]. The proton spectrum of **3f** has shown two diastereoisomers presented in their enol-forms (Figure 2) in a ratio of 1:0.65. Most probably the appearance of two isomers is defined by the volume and the configuration of the pyran ring in **3f** might be close to planar. Definitely the most stable enol-form makes the structure flat; moreover, it might be the driving force for flattening the radical participating in the homodimerization step. This process determines a possibility for coupling from both sides of the intermediate which produces a *meso*-form (4S,4′R) **3f_A_** and the pair of (4S,4′S)- and (4R,4′R)-stereoisomers **3f_B_** presented in Figure 2, the last are not distinguishable in NMR spectra.

The homodimerization of coumarin **1g** is presented by resonances for different stereoisomers in the proton NMR spectrum of the crude reaction mixture. The keto-forms have similar chemical shifts for H-3 and H-4 protons as in the other biscoumarins **3** and we could assign them as *meso*-forms. The signals for the enol-forms are several with doublets for the H-4 and H-4′ protons where the observed coupling constants of 9.3 Hz is associated with an angle close to 0 degrees between them. We distinguished the keto- and enol-forms by carrying out thin-layer chromatography of the reaction mixture using *n*-hexane and ethyl acetate as mobile phase; however, the separation by column chromatography did not lead to isolation of individual stereoisomers. Only the *meso*-isomer **3g** (Figure 2) was precipitated from the reaction mixture and its proton and carbon chemical shifts are listed in the Experimental part. From the same proton spectrum, the signals for the enol-form of **3g** are assigned and are also presented.

The tetrahydrobiscoumarin of **1h** is characterized as a mixture of two stereoisomers, in approximately equal ratio 1:1.1, on the basis of observed signals in the proton NMR spectrum of the crude reaction mixture. The two compounds **3h_A_** and **3h_B_** can be crystalized with minor presence of the other isomer using the appropriate solvents (see Section 4.).

The dimer from 3-benzoylcoumarin **1i** is presented by one *meso*-isomer **3i** which is differentiated from all the other products with low solubility in various organic solvents. A notable observation from the proton spectrum is the higher frequency for the H-3 and H-4 protons, compared to the values in the other homodimers **3**, resulted from the magnetic anisotropy of the carbonyl group.

The reaction with coumarin **1j** was carried out applying Method B and the desired product **3j** was isolated after column chromatography as aci-enol form (Figure 2). Such tautomers of nitro compounds are not frequently detected by spectroscopy or reported in the literature. Previously, we reported [40] the 1,4-addition of nitromethane to 3-nitrocoumarin that undergoes nitro- to aci-nitro-form in solution. Here the disposition of the substituents around the C3-C4 bond have similar configuration and it is not surprising to assign an enol-structure. Moreover, chelation between aci-form of nitro group and the C=O from the lactone ring could additionally stabilize this configuration. The proton spectrum of **3j** in CDCl_3_ represents a broad signal at 4.24 ppm for the enol and disappeared in deuterated methanol which proves the participation of the proton in chemical exchange process. Another interesting fact is the anisotropic effect of the nitro group on H-4, shifting it to a higher frequency of 6.71 ppm. If we compare the data from ^13^C NMR of **3j** and the other enol forms from the current investigation, **3f** and **3g**, the chemical shifts of C-3 and C-4 nuclei changed from around 92 and 46 ppm to higher frequency 131 and 110.9 ppm, respectively. Moreover, the data from ^1^H-^15^N HMBC show a signal at 50.474 ppm for the nitrogen nuclei as a long range correlation with the H4 which is another fact supporting the proposed structure.

## 3. Conclusions

The current investigations present a new method for faster and simple preparation of 3,3′,4,4′-tetrahydro-3,3′-disubstituted-4,4′-biscoumarins when an organozinc reagent is used. The ultrasound promoted reaction offers a simple experimental setup and reproducibility of the results. The homodimers are isolated with the highest yields reported in the literature about such compounds. Unlike previous synthetic procedures, our dimerization conditions are applicable for coumarins with electron-withdrawing groups in third position. We hypothesize that the formed radical, stabilized by the substituent and assisted by the organozinc compound, plays a major role on the dimerization mechanism. The structures of tetrahydrocoumarins are characterized by IR, NMR, MS, and X-ray data and the main product was assigned to be the *meso*-stereoisomer.

## 4. Experimental Section

### 4.1. Materials

Melting points were determined with a Kofler hot-stage apparatus (Reichert Technologies, New York, NY, USA) and are used without correction. The IR spectra were recorded with a Specord IR 71, IR 75 spectrophotometer (Carl Zeiss, 73447 Oberkochen, Germany). ^1^Н-, ^13^С-, and ^31^P-NMR spectra were recorded on a Bruker Avance III 500 spectrometer (Bruker BioSpin GmbH, Rheinstetten, Germany) (at 500 MHz for ^1^H, 125.7 MHz for ^13^C and 202.4 MHz for ^31^P, respectively). Chemical shifts are given in ppm downfield from tetramethylsilane as internal standard with CDCl_3_, DMSO, and CD_3_OD as solvents. ^31^P-NMR spectra were recorded with 85% H_3_PO_4_ as an external standard. Analyses were carried out on Q Exactive^®^ mass analyzer equipped with TurboFlow^®^ LC system and IonMax II^®^ electrospray ionization module (ThermoScientific Co., Waltham, MA, USA). Data acquisition and processing were carried out using the XCalibur^®^ 4.2 software package (ThetmoScientific Co., Waltham, MA, USA). Chromatographic conditions: Column: Syncronis C18, 1.7 μm (100 × 2.1 mm) (ThetmoScientific Co., Waltham, MA, USA). Mobile phase: A = 0.1 % formic acid in water and B = 0.1% formic acid in acetonitrile with flow rate: 300 μL/min. Mass spectrometric conditions: Full-scan spectra over the *m/z* range 120 to 1200 were acquired in positive ion mode at resolution settings of 140,000 (*m/z* = 200). All MS parameters were optimized for sensitivity to the target analyzes using the instrument control software program. Operating parameters: spray voltage 4.0 kV, sheath gas flow rate 32, auxiliary gas flow rate 10, spare gas flow rate 3, capillary temperature 280 °C, probe heater temperature 320 °C, and S-lens RF level 50. Data acquisition and processing were carried out with Xcalibur 4.2^®^ software package (ThetmoScientific Co., Waltham, MA, USA). Ultrasonic irradiation was performed in an ultrasonic cleaner with a frequency of 20 kHz and power 250 W. Reactions were monitored by TLC on silica gel 60 F_254_. Column chromatography was carried out on silica gel (Merck 0.043–0.063 mm) (Merck, Kenilworth, NJ, USA) using as eluent *n*-hexane/EtOAc mixture with increasing polarity. Elemental analyses of *C*, *H*, and *N* were carried out in the Laboratory of Elemental Analysis at the Department of Organic Chemistry and Pharmacognosy, University of Sofia, Bulgaria. The X-ray analysis was performed on Bruker Apex-II CCD diffractometer at the Laboratory of Molecular Spectroscopy of Structural Analysis, University of Sofia, Bulgaria. The crystal was kept at 300.15 K during data collection. Using Olex2 [41], the structure was solved with the SIR2004 [42] structure solution program using Direct Methods and refined with the ShelXL [43] refinement package using “Least Squares” minimization.

All chemical reagents were purchased from Merck and Sigma Aldrich (Taufkirchen, Germany). The starting 3-substitueted 2-oxo-2*H*-1-benzopyrans **1** were prepared according to a procedures reported by us [44].

### 4.2. General Methods

Method A: A mixture of **1** (0.001 mol), Zn (0.183 g, 0.0028 mol), (ClCH_2_CO)_2_O (0.410 g, 0.0024 mol) in Et_2_O/THF (10 mL/7 mL), and a catalytic amount of I_2_ was sonicated until the coumarin **1** was consumed (TLC-monitoring). The reaction mixture was poured onto a 2 N solution of hydrochloric acid and ice, extracted with chloroform (3 × 20 mL), and the organic extracts were washed several times with saturated solution of NaHCO_3_ and then dried with anhydrous sodium sulfate. After the evaporation of the solvent, to the residue were added 3 mL Et_2_O and 1 mL acetone and the resulting mixture was left in a fridge overnight. Compound **3** was obtained as a solid. After the filtration of the crystals, the solvent of the mother liquor was evaporated and the residue was purified by column chromatography using *n*-hexane/EtOAc as an eluent system.

Method B: Performed according to Method A, however the used metallic Zn powder was 0.366 g (0.0056 mol).

Method C: A mixture of **1a** (0.282 g, 0.001 mol), Zn (0.222 g, 0.0034 mol), (ClCH_2_CO)_2_O (0.256 g, 0.0015 mol) in Et_2_O/THF (10 mL/7 mL), and a catalytic amount of I_2_ was sonicated for 24 h.

*Tetraethyl (2,2′-dioxo-[4,4′-bichroman]-3,3′-diyl)bis(phosphonate)*, **3a**—Method B. The product was isolated from Et_2_O/acetone: 0.251 g, 89% white crystals, m.p. = 225–228 °C. IR (nujol): ν = 1775, 1060, 1035, cm^−1^. ^1^H NMR (500 MHz, DMSO) δ = 7.45 (dd, *J* = 7.0, 1.0 Hz, 2H, H-5/H-5′), 7.27 (td, *J* = 7.4, 1.1 Hz, 2H, H-7/H-7′), 7.02 (td, *J* = 6.5, 0.9 Hz, 2H, H-8/H-8′), 6.76 (dd, *J* = 7.9, 0.9 Hz, 2H, H-6/H-6′), 4.53 (d, *J* = 28.3, 2H, H-3/H-3′), 4.01–4.14 (m, 4H, POCH_2_CH_3_), 3.95 (d, *J* = 15, 2H, H-4/H-4′), 3.62–3.71 (m, 2H, POCH_2_CH_3_), 3.35–3.43 (m, 2H, POCH_2_CH_3_), 1.21 (t, *J* = 7.0 Hz, 6H, POCH_2_CH_3_), 0.72 (t, *J* = 7.1 Hz, 6H, POCH_2_CH_3_); ^13^C NMR (125.7 MHz, DMSO) δ = 163.2 (d, *J* = 6.3 Hz, C-2/C-2′), 151.9 (s, C-8a/C-8a′), 130.9 (s, C-7/C-7′), 130.2 (s, C-5/C-5′), 124.6 (s, C-8/C-8′), 119.2 (s, C-4a/C-4a′), 116.3 (s. C-6/C-6′), 63.2 (d, *J* = 6.3 Hz, POCH_2_CH_3_), 62.6 (d, *J* = 7.2 Hz, POCH_2_CH_3_), 43.3 (d, *J* = 122.1 Hz, C-3/C-3′), 41.7 (d, *J* = 3.5 Hz, C-4/C-4′), 16.5 (d, *J* = 6.5 Hz, POCH_2_CH_3_), 16.0 (d, *J* = 6.4 Hz, POCH_2_CH_3_); ^31^P NMR (202.4 MHz, DMSO): δ = 20.75 (s). HRMS (FTMS+p ESI) *m/z* calculated for C_26_H_32_O_10_P_2_ [M]^+^ 567.1549 found 567.1528.

*Tetramethyl (2,2′-dioxo-[4,4′-bichroman]-3,3′-diyl)bis(phosphonate)*, **3b**—Method B. The product was isolated from Et_2_O/acetone: 0.122 g, 61%, white crystals, m.p. = 265–266 °C. IR (CHCl_3_): ν = 1760, 1050, 1035 cm^−1^. ^1^H NMR (500 MHz, DMSO) δ = 7.45 (td, *J* = 6.5, 1.5 Hz, 2H, H-5/H-5′), 7.23 (td, *J* = 6.5, 1.0 Hz, 2H, H-7/H-7′), 7.12 (dd, *J* = 8.2, 0.8 Hz, 2H, H-8/H-8′), 6.76 (dd, *J* = 7.6, 1.4 Hz, 2H, H-6/H-6′), 3.57 (d, *J* = 11.3, 3H, POCH_3_), 3.55–3.57 (m, 2H, H-3/H-3′), 3.52 (d, *J* = 26.5, 2H, H-4/H-4′); ^13^C NMR (125.7 MHz, DMSO) δ = 163.1 (d, *J* = 3.3 Hz, C-2/C-2′), 150.9 (s, C-8a/C-8a′), 130.3 (s, C-7/C-7′), 129.1 (s, C-5/C-5′), 125.1 (s, C-8/C-8′), 119.3 (s, C-4a/C-4a′), 116.8 (s, C-6/C-6′), 53.4 (d, *J* = 6.9 Hz, POCH_3_), 53.3 (d, *J* = 6.9 Hz, POCH_3_), 42.9 (dd, *J* = 134.5, 1.3 Hz, C-3/C-3′), 41.7 (dd, *J* = 19, 3.8 Hz, C-4/C-4′), ^31^P NMR (202.4 MHz, DMSO): δ = 20.19 (s). Anal. Calcd for C_22_H_24_O_10_P_2_: C,51.55; H,5.12. Found: C, 51.75; H, 5.28. HRMS (FTMS-p ESI) *m/z* calculated for C_22_H_24_O_10_P_2_ [M]^−^ 509.0767 found 509.0756.

*Diethyl 2,2′-dioxo-[4,4′-bichroman]-3,3′-dicarboxylate*, **3c**—Method B. The product was isolated from Et_2_O/acetone: 0.159 g, 73%, white crystals, m.p. = 212–214 °C. IR (nujol): ν = 1795, 1710, 1460 cm^−1^. ^1^H NMR (500 MHz, CDCl_3_) δ = 7.41 (td, *J* = 7.5, 1.8 Hz, 2H, H-7/H-7′), 7.26 (td, *J* = 7.4, 1.7 Hz, 2H, H-5/H-5′), 7.23 (dq, *J* = 14.8, 7.4, 1.1 Hz, 2H, H-8/H-8′), 7.19 (dd, *J* = 8.1, 0.8 Hz, 2H, H-6/H-6′), 4.02–3.91 (m, 6H, COOCH_2_CH_3_), 3.78 (s, 2H, H-3/H-3′), 3.29 (s, 2H, H-4/H-4‘), 0.94 (t, 6H, COOCH_2_CH_3_); ^13^C NMR (125.7 MHz, CDCl_3_) δ = 165.9 (s, COOCH_2_CH_3_), 163.9 (s, C-2/C-2′), 150.8 (s, C-8a/C-8a’), 130.4 (s, C-7/C-7′), 130.1 (s, C-5/C-5′), 125.4 (s, C-8/C-8′), 120.6 (s, C-4a/C-4a’), 117.9 (s, C-6/C-6′), 62.5 (s, COOCH_2_CH_3_), 49.9 (s, C-4/C-4′), 42.3 (s, C-3/C-3′), 13.7 (s, COOCH_2_CH_3_). Anal. Calcd for C_24_H_22_O_8_: C,65.74; H,5.06. Found: C, 65.70; H, 5.03. HRMS (FTMS+p ESI) *m/z* calculated for C_24_H_22_O_8_ [M + H + NH_3_]^+^ 456.1658 found 456.1649.

*Dimethyl 2,2′-dioxo-[4,4′-bichroman]-3,3′-dicarboxylate*, **3d**—Method B. The product was isolated from Et_2_O/acetone: 0.194g, 73%, white crystals, m.p. = 225–226 °C. IR (nujol): ν = 1795, 1725, 1455 cm^−1^. ^1^H NMR (500 MHz, CDCl_3_) δ = 7.41 (ddd, *J* = 8.1, 7.0, 2.0 Hz, 2H, H-7/H-7′), 7.26 (td, *J* = 5.5, 2.0 Hz, 2H, H-5/H-5′), 7.24 (dq, *J* = 14.5, 6.4, 1.0 Hz, 2H, H-8/H-8′), 7.19 (dd, *J* = 8.1, 0.9 Hz, 2H, H-6/H-6′), 3.82 (s, 2H, H-3/H-3′), 3.52 (s, 6H, COOCH_3_), 3.29 (s, 2H, H-4/H-4′); ^13^C NMR (125.7 MHz, CDCl_3_) δ = 166.3 (s, COOCH_3_), 163.6 (s, C-2/C-2′), 150.6 (s, C-8a/C-8a′), 130.4 (s, C-7/C-7′), 130.1 (s, C-5/C-5′), 125.5 (s, C-8/C-8′), 120.4 (s, C-4a/C-4a′), 117.9 (s, C-6/C-6′), 49.7 (s, C-4/C-4′), 42.0 (s, C-3/C-3′), 53.3 (s, COOCH_3_). HRMS (FTMS-p ESI) *m/z* calculated for C_22_H_18_O_8_ [M]^−^ 409.0924 found 409.0918.

*Diphenyl 2,2′-dioxo-[4,4′-bichroman]-3,3′-dicarboxylate*, **3e**—Method B. The product was isolated from Et_2_O/acetone: 0.176 g, 66%, white crystals, m.p. = 180–183 °C. IR (nujol): ν = 1775, 1745, 1460 cm^−1^. ^1^H NMR (500 MHz, CDCl_3_) δ = 7.49 (td, *J* = 7.5, 1.6 Hz, 2H, aromatic), 7.39 (dd, *J* = 7.4, 1.5 Hz, 2H, H-5/H-5′), 7.33 (td, *J* = 6.3, 1.0 Hz, 2H, H-4′′ and aromatic), 7.29 (dd, *J* = 8.0, 0.8 Hz, 2H, H-4′′ and aromatic), 7.22–7.25 (m, 4H, COOPh), 7.15–7.18 (m, 2H, aromatic), 6.60 (dd, *J* = 8.2, 1.0 Hz, 4H, COOPh), 4.07 (s, 2H, H-3/H-3′), 3.49 (s, 2H, H-4/H-4′); ^13^C NMR (125.7 MHz, CDCl_3_) δ = 164.8 (s, COOPh), 163.3 (s, C-2/C-2′), 150.9 (s, C-8a/C-8a′), 149.6 (s, C-1′′ from COOPh), 130.8 (s, C-7/C-7′), 130.2 (s, C-5/C-5′), 129.5 (s, C-2′′ and C-6′′ from COOPh), 126.6 (s, C-4′′ from COOPh), 125.8 (s, C-8/C-8′), 120.7 (s, C-3′′ and C-5′′ from COOPh), 120.4 (s, C-4a/C-4a′), 118.2 (s, C-6/C-6′), 50.0 (s, C-4/C-4′), 42.6 (s, C-3/C-3′). HRMS (FTMS-p ESI) *m/z* calculated for C_32_H_22_O_8_ [M]^−^ 533.1236 found 533.1223.

*3,3′-Diacetyl-[4,4′-bichroman]-2,2′-dione*, **3f**—Method B. The product was isolated as two isomers **3f_A_** and **3f_B_** from Et_2_O/acetone: 0.173 g, 92%, white crystals, m.p. = 196–198 °C. IR (nujol): ν = 1595, 1645, 1450 cm^−1^.

**3f_A_** (major isomer): ^1^H NMR (500 MHz, CDCl_3_) δ = 12.95 (d, *J* = 0.6 Hz, 2H, enol form =C(CH_3_)OH), 7.34 (td, *J* = 8.0, 1.5 Hz, 2H, H-7/H-7′), 7.13 (td, *J* = 7.4, 1.1 Hz, 2H, H-8/H-8′), 7.08 (dd, *J* = 8.4, 1.0 Hz, 2H, H-6/H-6′), 6.86 (td, *J* = 8.2, 1.5 Hz, 2H, H-5/H-5′), 3.49 (s, 2H, H-4/H-4′), 1.41 (s, 6H, CH_3_); ^13^C NMR (125.7 MHz, CDCl_3_) δ = 178.3 (s, enol form =C(CH_3_)OH), 169.7 (s, C-2/C-2′), 150.9 (s, C-8a/C-8a′), 129.4 (s, C-7/C-7′), 129.1 (s, C-5/C-5′), 124.6 (s, C-8/C-8′), 122.9 (s, C-4a/C-4a′), 116.8 (s, C-6/C-6′), 93.9 (s, C-3/C-3′), 45.1 (s, C-4/C-4′), 17.6 (s, CH_3_). HRMS (FTMS+p ESI) *m/z* calculated for C_22_H_18_O_6_ [M + H + NH_3_]^+^ 396.1447 found 396.1522.

**3f_B_** (minor isomer): ^1^H NMR (500 MHz, CDCl_3_) δ = 13.19 (d, *J* = 0.5 Hz, 2H, enol form =C(CH_3_)OH), 7.29 (td, *J* = 8.0, 1.5 Hz, 2H, H-7/H-7′), 7.05 (d, *J* = 6.4 Hz, 2H, H-8/H-8′), 7.03 (dd, *J* = 8.1, 0.9 Hz, 2H, H-6/H-6′), 6.86 (td, *J* = 8.2, 1.5 Hz, 2H, H-5/H-5′), 3.73 (s, 2H, H-4/H-4′), 1.69 (s, 6H, CH_3_); ^13^C NMR (125.7 MHz, CDCl_3_) δ = 177.5 (s, enol form =C(CH_3_)OH), 168.9 (s, C-2/C-2′), 151.0 (s, C-8a/C-8a′), 129.2 (s, C-7/C-7′), 128.9 (s, C-5/C-5′), 124.7 (s, C-8/C-8′), 122.6 (s, C-4a/C-4a′), 116.9 (s, C-6/C-6′), 93.0 (s, C-3/C-3′), 46.4 (s, C-4/C-4′), 18.1 (s, CH_3_). HRMS (FTMS+p ESI) *m/z* calculated for C_22_H_18_O_6_ [M + H + NH_3_]^+^ 396.1447 found 396.152.

*3,3′-Diisobutyryl-[4,4′-bichroman]-2,2′-dione*, **3g**—Method B. The product was isolated in meso- and enol-forms from Et_2_O/acetone: 0.186 g, 86%, white crystals, m.p. = 256−257 °C.

**3g meso-form**–isolated from Et_2_O/acetone 0.036 g: IR (nujol): ν = 1750, 1700, 1450 cm^−1 1^H NMR (500 MHz, CDCl_3_) δ = 7.39 (ddd, *J* = 8.1, 6.9, 2.0 Hz, 2H, aromatic), 7.24–7.27 (m, 4H, aromatic), 7.16 (d, *J* = 8.0 Hz, 2H, aromatic), 3.94 (s, 2H, H-3/H-3′), 3.26 (s, 2H, H-4/H-4′), 2.62–2.7 (sept, 2H, CH(CH_3_)_2_), 0.91 (d, *J* = 6.9 Hz, 6H, CH_3_), 0.78 (d, *J* = 6.9 Hz, 6H, CH_3_); ^13^C NMR (125.7 MHz, CDCl_3_) δ = 204.6 (s, C=0), 165.1 (s, C-2/C-2′), 150.4 (s, C-8a/C-8a′), 130.3 (s, C-7/C-7′), 130.0 (s, C-5/C-5′), 125.5 (s, C-8/C-8′), 120.9 (s, C-4a/C-4a′), 117.8 (s, C-6/C-6′), 55.8 (s, C-3/C-3′), 45.1 (s, C-4/C-4′), 38.6 (s, CH(CH_3_)_2_), 18.1 (s, CH_3_), 17.9 (s, CH_3_). HRMS (FTMS+p ESI) *m/z* calculated for C_26_H_26_O_6_ [M + H + NH_3_]^+^ 452.2073 found 452.2159.

**3g enol form**: ^1^H NMR (500 MHz, CDCl_3_) δ = 13.05 (d, 1H, *J* = 1.5 Hz, =C(CH)OH), 7.31 (td, *J* = 8.1, 1.5 Hz, 2H, aromatic), 7.19–7.22 (m, 2H, aromatic), 7.11–7.13 (m, 2H, aromatic), 7.02 (dd, *J* = 8.2, 0.9, 2H, aromatic), 3.66 (d, 1H, *J* = 9.3 Hz, H-4), 3.26 (d, 1H, *J* = 9.3 Hz, H-4′), 2.56–2.62 (sept, 2H, CH(CH_3_)_2_), 0.97 (d, *J* = 6.9 Hz, CH_3_), 0.88 (d, *J* = 6.9 Hz, CH_3_), 0.77 (d, *J* = 6.9 Hz, CH_3_), 0.45 (d, *J* = 6.9 Hz, CH_3_); ^13^C NMR (125.7 MHz, CDCl_3_) δ = 170.1 (s, =C(CH)OH), 165.1 (s, C-2/C-2′), 150.8 (s, C-8a/C-8a′), 129.8 (s, C-7/C-7′), 139.5 (s, C-5/C-5′), 124.9 (s, C-8/C-8′), 119.1 (s, C-4a/C-4a′), 117.1 (s, C-6/C-6′), 92.1 (s, C-3/C-3′), 45.4 (s, C-4/C-4′), 38.7 (s, CH(CH_3_)_2_), 18.6 (s, CH_3_), 18.3 (s, CH_3_). HRMS (FTMS+p ESI) *m/z* calculated for C_26_H_26_O_6_ [M + H + NH_3_]^+^ 452.2073 found 452.2159.

*3,3′-Dipivaloyl-[4,4′-bichroman]-2,2′-dione*, **3h**—Method B. The product was isolated as two isomers **3h_A_** and **3h_B_** from Et_2_O/acetone: 0.212 g, 92%, white crystals, m.p. = 308–310 °C. IR (nujol): ν = 1745, 1695, 1450 cm^−1^.

**3h_A_**: ^1^H NMR (500 MHz, CDCl_3_) δ = 7.30 (td, *J* = 8.1, 1.5 Hz, 2H, aromatic), 7.11 (dd, *J* = 8.1, 0.9 Hz, 2H, aromatic), 6.91 (td, *J* = 7.5, 1.1 Hz, 2H, aromatic), 6.48 (dd, *J* = 7.5, 1.4 Hz, 2H, H-5/H-5′), 4.52 (s, 2H, H-3/H-3′), 3.19 (s, 2H, H-4/H-4′), 1.13 (s, 18H, CH_3_); ^13^C NMR (125.7 MHz, CDCl_3_) δ = 206.2 (s, C=O), 164.1 (s, C-2/C-2′), 151.2 (s, C-8a/C-8a′), 129.9 (s, C-7/C-7′), 129.6 (s, C-5/C-5′), 124.6 (s, C-8/C-8′), 119.4 (s, C-4a/C-4a′), 116.8 (s, C-6/C-6′), 50.4 (s, C-3/C-3′), 46.1 (s, C(CH_3_)_3_), 44.7 (s, C-4/C-4′), 26.3 (s, CH_3_). HRMS (FTMS+p ESI) *m/z* calculated for C_28_H_30_O_6_ [M + H + NH_3_]^+^ 480.2382 found 480.2481.

**3h_B_**: ^1^H NMR (500 MHz, CDCl_3_) δ = 7.46 (dd, *J* = 7.6, 1.6 Hz, 1H, aromatic), 7.44 (dd, *J* = 7.6, 1.6 Hz, 1H, aromatic), 7.28 (td, *J* = 7.4, 1.1 Hz, 2H, aromatic), 7.22 (dd, *J* = 6.1, 1.5 Hz, 2H, H-5/H-5′), 7.21 (dd, *J* = 6.3, 1.0 Hz, 2H, aromatic), 4.41 (s, 2H, H-3/H-3′), 2.97 (s, 2H, H-4/H-4′), 0.88 (s, 18H, CH_3_); ^13^C NMR (125.7 MHz, CDCl_3_) δ = 206.4 (s, C=O), 164.6 (s, C-2/C-2′), 151.2 (s, C-8a/C-8a′), 130.4 (s, C-7/C-7′), 130.0 (s, C-5/C-5′), 125.2 (s, C-8/C-8′), 119.7 (s, C-4a/C-4a′), 117.5 (s, C-6/C-6′), 50.8 (s, C-3/C-3′), 45.7 (s, C(CH_3_)_3_), 43.3 (s, C-4/C-4′), 25.9 (s, CH_3_). HRMS (FTMS+p ESI) *m/z* calculated for C_28_H_30_O_6_ [M + H + NH_3_]^+^ 480.2382 found 480.2481.

*3,3′-Dibenzoyl-[4,4′-bichroman]-2,2′-dione*, **3i**—Method B. The product was isolated from Et_2_O/acetone: 0.209 g, 84%, white crystals, m.p. = 188–192 °C. IR (nujol): ν = 1775, 1675, 1450, 1445 cm^−1^. ^1^H NMR (500 MHz, DMSO) δ = 8.07 (d, *J* = 7.8 Hz, 4H, H-2′′ and H-6′′), 7.83 (t, *J* = 7.2 Hz, 2H, H-4′′), 7.67 (d, *J* = 7.2 Hz, 4H, H-3′′ and H-5′′), 7.29 (t, *J* = 8.1 Hz, 2H, H-7/H-7′), 7.26 (d, *J* = 7.4 Hz, 2H, H-5/H-5′), 6.98 (t, *J* = 7.1 Hz, 2H, H-8/H-8′), 6.89 (d, *J* = 8.1 Hz, 2H, H-6/H-6′), 5.81 (s, 2H, H-3/H-3′), 4.09 (s, 2H, H-4/H-4′); ^13^C NMR (125.7 MHz, DMSO) δ = 195.2 (s, C=O), 165.2 (s, C-2/C-2′), 151.5 (s, C-8a/C-8a′), 135.3 (s, C-4′′), 133.6 (s, C-1′′), 130.9 (s, C-7/C-7′), 130.5 (s, C-5/C-5′), 129.7 (s, C-2′′ and C-6′′), 129.6 (s, C-3′′ and C-5′′), 124.7 (s, C-8/C-8′), 118.2 (s, C-4a/C-4a′), 116.7 (s, C-6/C-6′), 53.8 (s, C-3/C-3′), 44.02 (s, C-4/C-4′). HRMS (FTMS+p ESI) *m/z* calculated for C_32_H_22_O_6_ [M + H + NH_3_]^+^ 520.1863 found 520.1760.

*3,3′-Dinitro-[4,4′-bichroman]-2,2′-dione*, **3j**—Method B. The product was purified by column chromatography using *n*-hexane/EtOAc: 0.085 g, 45%, pale yellow crystals, m.p. = 131–133 °C. IR (nujol): ν = 3410, 3320, 1695, 1630, 1590, 1445 cm^−1^.

^1^H NMR (500 MHz, CDCl_3_) δ = 7.31–7.28 (dd, *J* = 8.1, 1.0 Hz, 2H, aromatic), 7.28–7.25 (m, 4H, aromatic), 7.22–7.19 (m, 2H, aromatic), 6.71 (s, 2H, H-4/H-4′), 4.24 (bs, =N(O)OH); ^13^C NMR (125.7 MHz, CDCl_3_) δ = 159.4 (s, C-2/C-2′), 149.1 (s, C-8a/C-8a′), 126.6 (s, C-7/C-7′), 125.1 (s, C-5/C-5′), 124.6 (s, C-8/C-8′), 116.2 (s, C-4a/C-4a′), 116.2 (s, C-6/C-6′), 131.9 (s, C-3/C-3′), 110.9 (s, C-4/C-4′).

^1^H NMR (500 MHz, CD_3_OD) δ = 7.26–7.24 (dd, *J* = 7.1, 1.2 Hz, 2H, aromatic), 7.17–7.14 (m, 1H, aromatic), 7.144–7.141 (m, 3H, aromatic), 7.12–7.09 (m, 2H, aromatic), 6.68 (s, 2H, H-4/H-4′); ^13^C NMR (125.7 MHz, CD_3_OD) δ = 159.7 (s, C-2/C-2′), 148.6 (s, C-8a/C-8a′), 132.9 (s, C-3/C-3′), 125.7 (s, C-7/C-7′), 124.7 (s, C-5/C-5′), 124.3 (s, C-8/C-8′), 121.8 (s, C-4a/C-4a′), 115.3 (s, C-6/C-6′), 109.4 (s, C-4/C-4′). ^1^H-^15^N-HMBC: 50.474 ppm. Anal. Calcd for C_18_H_12_O_8_N_2_: C, 56.26; H, 3.15; N, 7.29. Found: C, 56.08; H, 3.07; N, 7.12.

## Figures and Tables

**Table 1:** Homodimerization of 3-dialkylphosphonocoumarins **1a**–**b** under ultrasound irradiation.

Product	Substituent	Metal	Reaction Conditions–1: Metal: (ClCH_2_CO)_2_O					
			*Method A* 1:2.8:2.4		*Method B* 1:5.6:2.4		*Method C* 1:3.4:1.5	
			Time [min]	Yield [%]	Time [min]	Yield [%]	Time [min]	Yield [%]
**3a**	P(O)(OEt)_2_	Mg	420	14	-	-	-	-
**3a**	P(O)(OEt)_2_	Zn	420	70	180	89	2040	30 ^a^
**3b**	P(O)(OMe)_2_	Zn	420	25 ^a^	200	61	-	-

^a^ There was not full conversion of **1a**.

**Table 2:** Solvents used for the homodimerization of 3-dialkylphosphonocoumarins **1a**–**b** (Method A).

Solvent	Reaction Time [min]	Yield
THF	120	Complex mixture
Et_2_O	420	N/A
THF:Et_2_O (3.5:5)	420	70%

**Table 3:** Homodimerization of 3-substituted coumarins **1c**–**n** under ultrasound reaction conditions.

Coumarin	Product	Y	Method A		Method B	
			Reaction time [min]	Yield [%]	Reaction time [min]	Yield [%]
**1c**	**3c**	COOEt	300	60	30	73
**1d**	**3d**	COOMe	240	51	30	73
**1e**	**3e**	COOPh	120	55	40	67
**1f**	**3f**	COMe	180	Complex mixture	10	92 ^a^
**1g**	**3g**	COiPr	1020	Partial conversion	15	86 ^a^
**1h**	**3h**	COtBu	390	71	180	92 ^a^
**1i**	**3i**	COPh	270	52	10	84
**1j**	**3j**	NO_2_	840	Complex mixture	90	45
**1k**	**3k**	CN	840	Complex mixture	270	Complex mixture
**1l**	**3l**	H	1020	N/A	120	N/A
**1m**	**3m**	Ph	1020	N/A	120	N/A
**1n**	**3n**	NHCOMe	720	N/A	-	-

^a^ Two or more isomers see Section 2.2. Spectroscopic data interpretation of compounds **3**.

**Table 4:** Mixed reactions on the coumarin dimerization.

Entry	Mixed Coumarin Reaction (Method B, Equimolar Ratio for the Coumarins)	Reaction Time [min]	Products
**1**	**1a:1m**	180	**3a:1m**
**2**	**1a:1c**	40	**1a:3c**
**3**	**1f:1i**	10	**3f:3i**
